# Exploring Immersive Multimodal Virtual Reality Training, Affective States, and Ecological Validity in Healthy Firefighters: Quasi-Experimental Study

**DOI:** 10.2196/53683

**Published:** 2024-10-24

**Authors:** Joana Oliveira, Joana Aires Dias, Rita Correia, Raquel Pinheiro, Vítor Reis, Daniela Sousa, Daniel Agostinho, Marco Simões, Miguel Castelo-Branco

**Affiliations:** 1 Coimbra Institute for Biomedical Imaging and Translational Research, Institute for Nuclear Sciences Applied to Health, University of Coimbra Coimbra Portugal; 2 Faculty of Medicine, University of Coimbra Coimbra Portugal; 3 National Fire Service School Sintra Portugal; 4 Center for Informatics and Systems of University of Coimbra, Faculty of Science and Technology, University of Coimbra Coimbra Portugal

**Keywords:** virtual reality, firefighter, training, posttraumatic stress disorder, PTSD, emotion, situational awareness, engagement, ecological validity, multivariate approach

## Abstract

**Background:**

Firefighters face stressful life-threatening events requiring fast decision-making. To better prepare for those situations, training is paramount, but errors in real-life training can be harmful. Virtual reality (VR) simulations provide the desired realism while enabling practice in a secure and controlled environment. Firefighters’ affective states are also crucial as they are a higher-risk group.

**Objective:**

To assess the impact on affective states of 2 simulated immersive experiences in a sample of healthy firefighters (before, during, and after the simulation), we pursued a multivariate approach comprising cognitive performance, situational awareness, depression, anxiety, stress, number of previous adverse events experienced, posttraumatic stress disorder (PTSD) severity, and emotions. The efficacy and ecological validity of an innovative VR haptic system were also tested, exploring its impact on performance.

**Methods:**

In collaboration with the Portuguese National Fire Service School, we exposed 22 healthy firefighters to 2 immersive scenarios using the FLAIM Trainer VR system (neutral and arousing scenarios) while recording physiological data in a quasi-experimental study. Baseline cognitive performance, depression, anxiety, stress, number of adverse events, and severity of PTSD symptoms were evaluated. Positive and negative affective states were measured before, between, and after each scenario. Situational awareness, sense of presence, ecological validity, engagement, and negative effects resulting from VR immersion were tested.

**Results:**

Baseline positive affect score was high (mean 32.4, SD 7.2) and increased after the VR tasks (partial η^2^=0.52; Greenhouse-Geisser *F*_1.82,32.78_=19.73; *P*<.001). Contrarily, mean negative affect score remained low (range 11.0-11.9) throughout the study (partial η^2^=0.02; Greenhouse-Geisser *F*_2.13,38.4_=0.39; *P*=.69). Participants’ feedback on the VR sense of presence was also positive, reporting a high sense of physical space (mean score 3.9, SD 0.8), ecological validity (mean score 3.8, SD 0.6), and engagement (mean score 3.8, SD 0.6). Engagement was related to the number of previously experienced adverse events (*r*=0.49; *P*=.02) and positive affect (after the last VR task; *r*=0.55; *P*=.02). Conversely, participants reported few negative effects (mean score 1.7, SD 0.6). The negative effects correlated positively with negative affect (after the last VR task; *r*=0.53; *P*=.03); and avoidance (*r*=0.73; *P*<.001), a PTSD symptom, controlling for relevant baseline variables. Performance related to situational awareness was positive (mean 46.4, SD 34.5), although no relation was found to metacognitively perceived situational awareness (*r*=–0.12; *P*=.59).

**Conclusions:**

We show that VR is an effective alternative to in-person training as it was considered ecologically valid and engaging while promoting positive emotions, with few negative repercussions. This corroborates the use of VR to test firefighters’ performance and situational awareness. Further research is needed to ascertain that firefighters with PTSD symptomatology are not negatively affected by VR. This study favors the use of VR training and provides new insights on its emotional and cognitive impact on the trainee.

## Introduction

### Background

Over the last few years, gamification and serious games applied to nongame settings have become a visible theme, promoting knowledge acquisition, user engagement, and strategic skills [1]. More precisely, virtual reality (VR) serious game training systems have been showing high potential for many purposes, especially in the professional instruction field [2,3]. Simulated situations under high stress levels have been gaining greater interest for some time. As an example, Keitel et al [4] examined endocrine and psychological stress responses in medical students during 2 simulated stress scenarios (a stress situation induced at a laboratory and a simulated emergency situation) compared to a rest condition.

For firefighters, this assumes special importance in relation to the idiosyncrasies of their strenuous everyday duties [2]. Their functions require a repetitive and continuous preparation to acquire and preserve skills and refresh expertise, which can be potentially facilitated through VR tools. These systems present direct advantages compared to traditional in-person training as a cost-effective and safer realistic alternative to perform dangerous or complex tasks and train decision-making and related abilities in secure environments, replicating similar conditions to those in real life with less time and consumption of resources or other constraints.

Furthermore, VR applications make available a plurality of training scenarios with high ecological validity [2]. Ecological validity can be defined as “the ability to generalize experimental results to different populations, situations, and variables” [5] and is related to the cues contained in an experiment that permit the generalization from the laboratory to real life. VR settings have been successfully used to increase the ecological validity of several experiments [6] as well as enhance participants’ daily activities, be it rehabilitation for older adults [7], improving cognitive function of children with attention-deficit/hyperactivity disorder [8], or simply shopping in a virtual supermarket [9].

The high ecological validity of VR scenarios allows for customized realistic immersive scenarios fit to the training purpose [10] without putting the workers’ lives or properties in danger [11]. These systems present several other advantages, namely, being an observer [12]; repeated practice; and promotion of the users’ engagement and motivation regarding the training tasks and, consequently, the retention of competencies and transference of knowledge [2,13]. VR experiences also encourage post–training session discussions and member collaboration [14]. This is critical because coordination and cooperation are essential between firefighters. Furthermore, VR technology provides user insights into simulated experiences and gains into real-life situations and can be applied to a wide range of personnel categories (eg, trainees and commanders).

As presented by Narciso et al [15], the ideal VR training system should reproduce with the best accuracy possible the real-world situations such that the user can be immersed in the virtual environment and perceive it as close to reality. Thus, the effectiveness of the VR system and its ability to mobilize the user are crucial points to consider regarding firefighting training. Moreover, these workers have to manage critical and dangerous incidents, operate in intense and stressful unpredictable conditions, face hazardous scenarios, deal with human suffering, and handle one’s own and colleagues’ emotions.

In the last years, some studies have applied simulated tasks [16] and VR systems using several devices and tools (eg, game engines using 3D graphics, software, and VR equipment) for the purpose of firefighter instruction [10,13,17-22]. Reis and Neves [23,24] presented a list of guidelines for simulation in training contexts using VR tools, namely, physical and psychological fidelity, interaction, immersive features, and realism. In addition, Saghafian et al [25] highlighted the importance of realism to increase the acceptance level of the users, effectiveness, and transfer of knowledge, as well as the role of emotions in VR contexts, for example, when using a VR fire extinguisher training tool for several companies from the industrial sector.

From our perspective, developments have been made in this area, with studies focused on VR for firefighting training purposes [15,23,26-28]. In a specific study by Narciso et al [29], virtual environments were used to investigate the effects of multisensory stimuli in 6 different methods—audiovisual or audiovisual combined with smell, or heat, or weight, or uniform, or mask, or a combination of these—on stress (using heart rate and self-report measures), fatigue, cybersickness, presence, and knowledge transfer. This study was conducted in the context of firefighter training and involved a sample of 91 individuals who were not firefighters. Another pilot study revealed the positive effects of resorting to VR tasks for firefighter training purposes, namely, in decision-making abilities during emergency actions [24].

Notwithstanding the existing literature, a clear gap remains in this area, including studies using multimodal experiments capable of integrating realistic immersive scenarios, neuropsychological measures, and biological parameters. The study by Andrews [16] called attention to the ecological and content validity issues of simulated emergency tasks for firefighters, aggregating findings from 60 protocols. The author also highlighted the lack of empirical studies concerning simulating tasks. Indeed, there is a clear need for in-depth ecological experiences, and VR systems might be a valid solution with high application potential.

Some explicit attempts have been made to increase realism in firefighter training [15,16,30]. For example, the study by Butler et al [30] has sought to integrate a multicomponent method including behavioral and biological parameters in a sample of incident commanders to investigate standard operating procedures and decision-making during simulated events. However, the technology used was scarcely immersive (the study used moving images and did not resort to VR technology). In addition, another study [31] investigated decision-making in a sample of Portuguese trainees who became certified for the job using a realistic VR system coupled to a haptic thermal device. The impact of VR was analyzed, but neither physiological measures nor the emotional and neuropsychological components were included.

Other studies have addressed firefighters’ situational awareness [12,32-34], defined as the “knowledge or how well the individual discriminates true (signal) from false (noise) information” [32]. However, the literature is still scarce in studying the potential of including this variable applied to firefighter training from a broader perspective, as argued by Chiu [12], namely, combined with VR methodology.

Despite the aforementioned body of work, further research is still needed regarding the effectiveness of VR training applied to firefighter instruction. For example, recently, in their study, Narciso et al [35] applied a heart rate variability measure and self-reported questionnaires to a sample of Portuguese firefighters in this VR context. Although the authors found promising results related to stress, sense of presence, cybersickness, and transference of knowledge, training in real environments showed superior results compared to the VR setting. This is unsurprising and does not preclude the use VR in transfer situations in which learning in real settings is not practical.

To successfully perform their duties, firefighters need flexibility for diverse tasks and operations and have to act immediately, make critical decisions in adverse conditions, deal with fatigue, and control their emotions. After interventions, it is central for their mental health to return to a homeostatically appropriate state to carry on with their lives. In this regard, a systematic review [36] found some mental health issues within firefighter groups, namely, posttraumatic stress disorder (PTSD), anxiety, and depression. Oliveira et al [37] also investigated this issue and replicated these findings.

Emotions play a crucial role in the mental health of firefighters [38]. Findings from Godfrey et al [39] revealed that difficulties in emotion regulation mechanisms were positively associated with the severity of PTSD symptomatology in a sample of firefighters and might be a valuable target considering the vulnerability of this population. Stanley et al [40] highlighted greater levels of distress tolerance as a key trait for firefighting, defined as a capability to deal with negative emotional and physical states. Specifically, firefighting is emotionally challenging work, being cognitively demanding and physically overloading, with implications for the decision-making process [41]. In this regard, Robinson et al [42] found some impairments in cognitive functions among nonfirefighter participants (namely, in the visual declarative memory and working memory domains immediately after the exposure and 20 minutes after) in response to the threat during a simulated firefighting emergency. Furthermore, a study found that shift workers have a higher propensity to show cognitive impairment due to sleep deprivation [43].

This state might affect fundamental cognitive abilities that are crucial to guarantee the success, effectiveness, and safety of firefighting operations. Previous research has indicated that firefighters require higher cognitive function during their hazardous activities [44,45]. Some examples of this higher-order cognition are attention (as a broad construct)—the ability to maintain focus over the time; vigilance—the capacity to maintain continuous attention to specific stimuli, helping in information discrimination; processing speed—the ability to acquire and process information rapidly and, consequently, execute [46]; analytical thinking—the ability to solve problems by virtue of understanding logical principles and assessing the evidence [47]; accurate decision-making ability as a complex process that includes making difficult choices, weighing potential risks and rewards, taking actions, and analyzing the effects [48,49]; working memory—the capacity to maintain information accessible while performing other complex tasks [50], involving temporary storage and manipulation of information; cognitive flexibility—the ability to respond readily, changing selectively according to the environment’s demands [51]; and awareness of the situation—the ability to continuously extract information from the surrounding dynamic environment [52], among other executive functions.

Considering the state of art, this study used an Australian VR realistic training system, the FLAIM Trainer, which uses immersive technology for firefighter training and learning. This simulator comprises several cutting-edge characteristics, including various immersive virtual fire scenarios, using a multisensory interface with several customized and haptic components (eg, heat suit, hose line, and a nozzle). For the purpose of this work, a multimodal approach was implemented using realistic training tasks combined with psychological and physiological measures, including evaluation of executive functions and firefighters’ performance regarding situational awareness.

### Objectives

The main goal of this work was to assess the impact of 2 simulated experiences on positive and negative emotions in a sample of healthy Portuguese firefighters pursuing a multivariate approach and, ultimately, investigate affective aspects of the immersive performance in the following two virtual environments: (1) a neutral scenario (exploration of the surrounding area of a rural residence, including preventive tasks) and (2) an arousing stressful scenario (extinction of a residential fire, including victim rescue actions). The relationship among cognitive performance; reported levels of anxiety, depression, and stress; PTSD severity; adverse events witnessed as a firefighter before the experiment; and affective states before, during, and after the VR task was also analyzed. Another aim was linked to this latter question and concerned evaluation of the impact of an innovative and haptic VR system (the FLAIM Trainer) on firefighter training and daily duties. The ecological effectiveness and the direct and indirect effects of these immersive scenarios were tested, including evaluation of firefighters’ situational awareness fit to firefighter demands. These aspects have direct implications for firefighting work and performance in relation to firefighter training and the role of emotion regulation mechanisms in the daily duties of these workers.

## Methods

### Overview

The sample comprised 22 healthy firefighters from several fire stations in Portugal (n=17, 77% from Lisbon and the Tagus Valley area) recruited in collaboration with the Portuguese National Fire Service School at Sintra (Portugal). The exclusion criteria were the presence of a severe psychiatric or physical condition affecting the brain and behavior—including PTSD, drug or alcohol addiction, significant vision and hearing problems, and pharmacological treatment with effects on brain mechanisms—and never having participated in firefighting campaigns. The Results section presents a detailed description of the sample.

### Protocol Procedure

**Figure 1:**
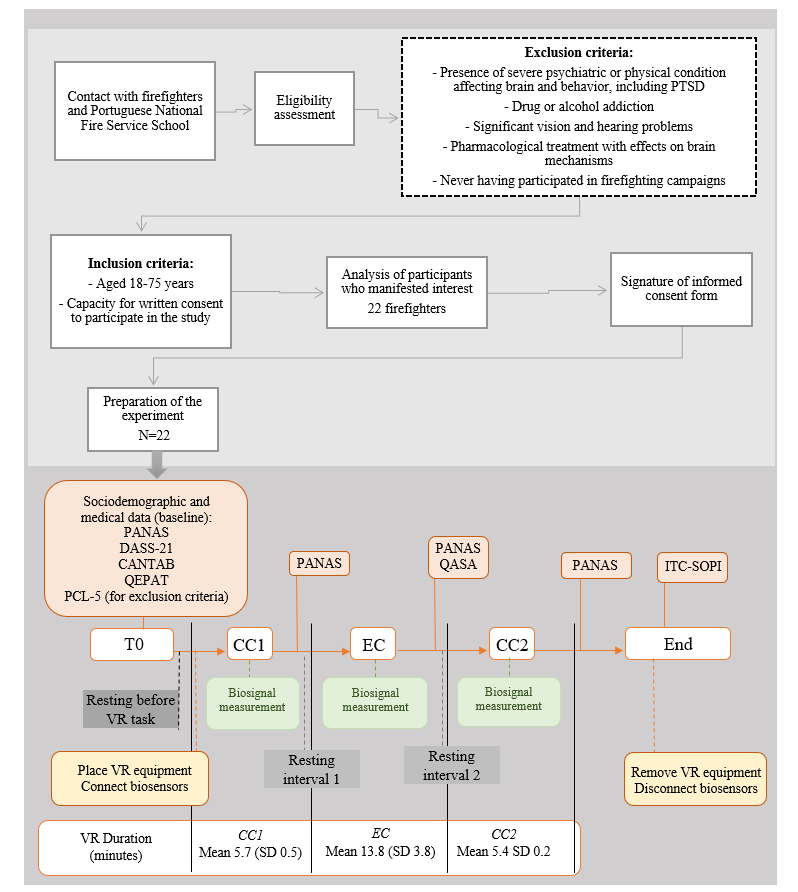
Diagram of the recruitment procedure and experimental sessions, including psychological assessment, virtual reality (VR) experiments, and biosignal recording. CANTAB: Cambridge Neuropsychological Test Automated Battery; CC1: control condition 1; CC2: control condition 2; DASS-21: 21-item Depression, Anxiety, and Stress Scales; EC: experimental condition; ITC-SOPI: ITC–Sense of Presence Inventory; PANAS: Positive and Negative Affect Schedule; PCL-5: Posttraumatic Stress Disorder Checklist for the Diagnostic and Statistical Manual of Mental Disorders, Fifth Edition; PTSD: posttraumatic stress disorder; QASA: Quantitative Analysis of Situation Awareness; QEPAT: Questionário de Exposição e Perturbação dos Acontecimentos Traumáticos (a Portuguese questionnaire related to the exposure to and disturbance of traumatic events); T0: baseline.

The experiment was conducted at the Portuguese National Fire Service School facilities at Sintra (Portugal), except in the case of 9% (2/22) of the participants, who performed the tasks at the Institute for Nuclear Sciences Applied to Health, University of Coimbra (Portugal), using a quasi-experimental study design. Data collection at the Portuguese National Fire Service School occurred during daytime with participants who were at the school attending a course or called up from nearby fire brigades to participate in the study. Data were also collected during daytime at the Institute for Nuclear Sciences Applied to Health with 9% (2/22) of the firefighters, who purposely went to the institute to participate in the study on a day off from work. Collection at both sites took place during the participants’ time off from work. All participants underwent an individual session, including a comprehensive semistructured interview regarding sociodemographic information and self-reported medical history followed by a psychological assessment comprising self-report measures and a battery of computerized tests to assess cognitive function. The total duration of the psychological assessment session was approximately 1 hour. Each participant was then conducted to a separate room to take part in the virtual experience session, including measurement of biosignals and filling out the self-report questionnaires immediately after undergoing each experimental scenario to evaluate their experience in the VR environment at various moments in time (Figure 1).

It was determined that the participants should complete the psychological assessment first and the VR experience afterward due to the consideration that performing the VR task first could elicit a particularly positive or negative mood and, thus, lead to mood-congruent memory recall bias [53,54] and inaccurate reports during the subsequent psychological assessment. To prevent the psychological assessment from having the opposite effect on the VR task performance, it was ensured that the participants had at least 25 minutes to rest between the psychological assessment and the VR task.

During all VR experiments (including during the filling out of the self-report measures), firefighters wore urban-standard fire combat equipment, comprising protective clothing and boots, to accurately simulate a real situation.

### Outcome measures

We collected sociodemographic and self-reported medical data, and the measurement instruments used were: (1) the Positive and Negative Affect Schedule (PANAS [55,56]); 2) 21-item Depression, Anxiety, and Stress Scales (DASS-21 [57,58]); 3) Questionário de Exposição e Perturbação dos Acontecimentos Traumáticos (QEPAT; a Portuguese questionnaire related to the exposure to and disturbance of traumatic events [59]); 4) Posttraumatic Stress Disorder Checklist for the Diagnostic and Statistical Manual of Mental Disorders, Fifth Edition (PCL-5; [60]; [61]); and 5) Cambridge Neuropsychological Test Automated Battery (CANTAB) [62].

The following selected tests were administered in this sequence before the VR tasks: motor screening task (MOT), spatial working memory (SWM) task (extended version), rapid visual information processing (RVP) task (3-target version), intra/extradimensional set shift (IED; lines-first-repeated version), the 6) Quantitative Analysis of Situation Awareness (QASA; instrument created for this study following the methodology proposed by Edgar et al [63]), and the 7) ITC–Sense of Presence Inventory (ITC-SOPI [64,65]).

More detailed information about the outcome measures used in this study and the internal consistency of the scales can be found in Multimedia Appendix 1.

All scales’ descriptive statistics, Cronbach α values, and detailed information about the duration of the experiment (conditions and resting intervals) can be found in Multimedia Appendix 2.

### VR Session: Setup

The FLAIM Trainer VR technology was designed for firefighter training and learning, presenting a wide range of simulated fire environments (150 available scenarios in 28 languages) [66], which faithfully reproduce dangerous situations usually faced in fire duties (eg, highway, national park, and warehouse fires) or rarer events (eg, fire in a petrol station or engine fire on an aircraft). The system was developed in an attempt to fit the needs regarding fire duties and integrated professional feedback; instructors can monitor in real time and review and track trainees’ performance. The simulator was acquired for research purposes within firefighter training.

Some case studies have been reported using the FLAIM Trainer VR system with positive results [67]. Higher levels of acceptance and usability of this VR system were found among 91 Brazilian military firefighters during a firefighting specialization course [68].

The simulator incorporates feedback and outcomes to assist firefighters in decision-making, along with a proprietary virtual fire behavior technology that mimics real fire. This technology aims to improve high-level skills such as risk assessment and dynamic thinking and practical abilities such as muscle memory, radio messaging, and hose and nozzle handling [69]. The hardware was designed considering user-friendliness and immersiveness. The user can explore and navigate the scenes freely, which is facilitated by a teleport mode, and interact with objects.

**Figure 2:**
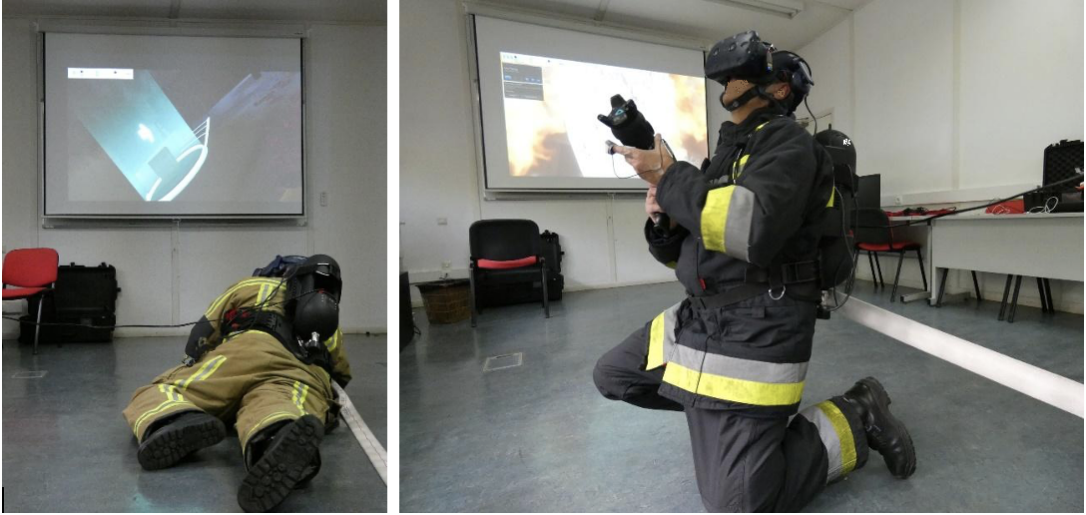
Two firefighters perform the virtual reality tasks (images from the Portuguese National Fire Service School).

The hardware setup includes customized components, namely, a self-contained breathing apparatus kit where the VR computer is housed with a built-in HTC Vive Pro VR headset, a half facemask and lung demand valve to capture respiration and reproduce sounds of breathing, and a thermal imaging camera, as well as a fire proximity heat suit technology and a mobile hose line apparatus with haptic technology reproducing a realistic jet and nozzle force; the pump pressure and the suppressant type can be controlled and customized at any time by the instructor. Support equipment includes an instructor tablet, HTC Vive Tracker Puck, charging systems, cables, peripherical hardware, tripods, tracking hardware with base stations (Vive Lighthouses), and Vive Controller [69,70]. In this experiment, the half facemask, thermal imaging camera, and heat suit were not used. Figure 2 shows 2 firefighters performing the VR tasks.

During the VR sessions, biosignals were recorded simultaneously through noninvasive superficial sensors to study autonomic arousal related to firefighters’ emotions and behavior and its impact on decision-making under stressful conditions. The parameters measured were electrodermal activity, photoplethysmography, electrocardiography, electroencephalography, and respiration. These data will be reported in a separate study.

**Figure 3:**
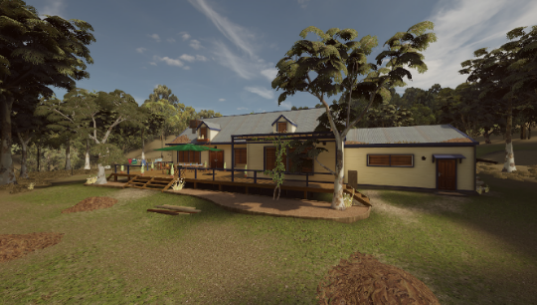
Scenario of control conditions 1 and 2 (provided by FLAIM).

The VR experience was divided into 3 moments with 2 different experimental scenarios. The experiment started with a brief tutorial for the user’s familiarization with the interaction with the VR system and the use of the hose and the teleport mode. Then, the neutral scenario (*Property Emergency Prepare* scenario; Figure 3) was presented—control condition 1. The firefighters were instructed to explore the scenario for 5 minutes and clean the terrain and the nature around a rural property as a prevention and safeguard measure against potential bushfire risks. In this environment, it was not possible to enter the house, but there were several elements outside with which the participant should interact (eg, cleaning inflammable debris from the nearby zone, ensuring the safety of the animals, and preparing the area for a potential fire).

**Figure 4:**
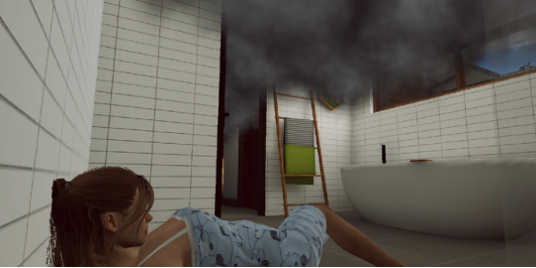
Scenario of the experimental condition (provided by FLAIM).

The experimental condition consisted of a residential fire in the first floor of a house inserted in an urban zone (Bedroom Fire scenario; Figure 4). The scenario ended when the firefighter extinguished the fire and rescued all human victims. The experiment ended with another immersion in the neutral scenario (Property Emergency Prepare scenario; Figure 3)—control condition 2—for 5 minutes, effectively acting as a postbaseline assessment. The firefighters were instructed to act as in a real situation during all conditions. A total of 14% (3/22) of the participants did not complete the last scenario (control condition 2) for health-related reasons (ie, shortness of breath caused by the physical demands of the experimental condition), due to technical issues (ie, system crashed due to lack of battery during the task), or because they did not wish to continue participating in the experiment.

Participants took a mean of 34.1 (SD 8.6) minutes to complete both the control and experimental conditions, also accounting for resting time between the conditions. While a 5-minute time limit was established for the control conditions, some participants needed more time to familiarize themselves with the VR headset and nozzle, so additional time was provided. This also had the aim of ensuring that the participants were engaged and motivated to perform the experimental condition. The first control condition had a mean completion time of 5.7 (SD 0.5) minutes, whereas the second control condition had a mean completion time of 5.4 (SD 0.2) minutes. No time limit was imposed for the experimental condition. Instead, it was determined that the participants had finished the experimental condition once they had completed the 2 objectives of putting out the fire and saving the victims or when they themselves decided to terminate the task. Even though participants were encouraged to complete the objectives, they could quit at any time. Only 5% (1/22) of the participants abandoned the experimental condition after not being able to complete it after 20.5 minutes. Furthermore, it was ensured that the participants had resting time between conditions to avoid fatigue, although the specific interval duration between conditions was determined by the participants. The mean resting times were 4.3 (SD 3.3) minutes between control condition 1 and the experimental condition and 6.5 (SD 1.6) minutes between the experimental condition and control condition 2.

### Statistical Analysis

Statistical analysis was performed using SPSS (version 28; IBM Corp), and a significance level of 0.05 was established. Detailed information about the statistical analysis performed can be found in Multimedia Appendix 1.

### Ethical Considerations

Participants provided written informed consent. This study was approved by the local research ethics committee of the Faculty of Medicine of the University of Coimbra (approval number: CE_Proc. CE-047/2020) and was guided by the Declaration of Helsinki of 1975 and its latest updates. All data were handled under rigorous protective measures in place to safeguard participant information; specifically, an alphanumeric code was assigned to all participants following a deidentification process to preserve participants’ confidentiality and privacy. No compensation was provided for participation in the study.

## Results

### Participants

Most participants were male (15/22, 68%), with a mean age of 39.1 (SD 9.7; median 42.0, range 23-56) years, and had completed the mandatory education in Portugal (secondary education; 17/22, 77%). In addition, 91% (20/22) and 82% (18/22) of the sample reported consuming alcohol and coffee, respectively, and 36% (8/22) smoked regularly. Regarding their history of psychological help, 59% (13/22) of the firefighters reported having sought psychological assistance in the past. Concerning their activity as firefighters, the sample was equally distributed between professional (12/22, 55%) and volunteer (10/22, 45%) firefighters working primarily in shifts (19/22, 86%) for a mean of 36.8 (SD 17.5; median 42.0, range 7-60) hours per week. The participants had worked for a mean of 18.9 (SD 10.6; median 18.0, range 4-40) years as firefighters. Detailed sociodemographic data can be found in Multimedia Appendix 3.

### Characterization of Psychological Measures

#### DASS-21 Results

Participants’ scores in the 3 scales of the DASS-21 were low as they all had normal depression scores (0 to 4), 9% (2/22) had mild anxiety scores (4 to 5), and only 5% (1/22) had mild stress scores (8 to 9). The sample mean was 1.1 (SD 1.3; median 1.0, range 0-4) for the depression scale, 1.2 (SD 1.5; median 0.5, range 0-5) for the anxiety scale, and 3.7 (the highest; SD 2.6; median 3.5, range 0-8) for the stress scale.

#### QEPAT Results

Regarding the number of adverse events in the context of firefighting activities, participants had experienced a mean of 31.9 (SD 4.5; median 33.0, range 23-40) event types out of 42. No additional events were described by any participants.

The most frequently experienced adverse events were participating in firefighting activities in which goods and properties were at risk (mean 2.7, SD 0.8; median 3.0, range 1-4) and aiding or seeing injured and/or fragile older adults (mean 2.7, SD 0.7; median 3.0, range 2-4), with 59% (13/22) of the sample indicating experiencing each event many times or frequently. In addition, 50% (11/22) of the sample indicated having seen or aided gravely injured adults many times or frequently (mean 2.6, SD 1.1; median 2.5, range 1-4).

While analyzing the level of disturbance, we considered only the events the participants had experienced. The events that obtained the highest mean disturbance score were witnessing the death or grave injury of a firefighter colleague while in active duty (mean 3.4, SD 0.7; median 4.0, range 2-4) and having aided a firefighter colleague who died or was gravely injured while in active duty (mean 3.2, SD 0.7; median 3.0, range 2-4). Both of these events were experienced by 41% (9/22) of the participants. One other event type that obtained a high disturbance score and was experienced by all but 1 participant (21/22, 95%) was hearing through radio communications that firefighter colleagues were in danger, injured, or dead (mean 3.3, SD 0.7; median 3.0, range 2-4). Of the events experienced by all participants, having seen or helped injured children was the one with the highest level of disturbance (mean 2.2, SD 0.9; median 2.0, range 1-4).

#### PCL-5 Results

None of the participants surpassed the PCL-5 full-scale thresholds or fulfilled the *Diagnostic and Statistical Manual of Mental Disorders, Fifth Edition*, criteria for possible PTSD diagnosis, yielding a sample mean score of 3.9 (SD 4.5; median 2.0, range 0-12). The subscales had similar low mean scores. In the Intrusion Symptoms and Avoidance subscales, the sample held a mean of 1.0 (SD 1.5; median 0.0, range 0-5) and 1.0 (SD 1.9; median 0.0, range 0-8), respectively, similar to that of the Negative Alterations in Cognitions and Mood (NACM; mean 1.1, SD 1.7; median 0.0, range 0-6) subscale and slightly lower scores on the Alterations in Arousal and Reactivity (AAR) subscale (mean 0.9, SD 1.3; median 0.0, range 0-4).

#### PANAS Results

Participants had low negative affect scores for all 4 moments of administration of the PANAS, ranging from a mean of 11.0 after both control conditions (SD 1.3 for control condition 1 and SD 1.8 for control condition 2) to 11.9 (SD 3.2) after the experimental condition. All mean scores were <12 although higher than the minimum score of 10 (*P*<.05 for all comparisons). Conversely, the scores on the positive affect scale were moderate to high. At the baseline measure (T0), the sample had a mean of 32.4 (SD 7.2), close to the middle point of 30 (Cohen *d*=0.33; t_21_=1.53; *P*=.14). The highest mean score was obtained for the measure after the second control condition (mean 40.7, SD 6.6), although it was lower than the maximum score of 50 on the scale (Cohen *d*=–1.42; t_18_=–6.18; *P*<.001). Detailed results can be found in Multimedia Appendix 4.

#### CANTAB Results

All participants excelled in the MOT, concluding the assessment trials with 0 mistakes.

In the SWM task (more detailed information about the outcome measures used in this study can be found in Multimedia Appendix 1), the mean was –0.6, measured in normalized *z* scores (SD 1.3; median –1.0, range −1.9 to 2.3), which is only slightly lower than the norm mean of *z*=0 (Cohen *d*=–0.47; t_21_=−2.22; *P*=.04). Only 18% (4/22) of the participants had values higher than the mean, with 14% (3/22) obtaining the highest possible score of *z*=2.3 (percentile 99). These 3 participants never made the mistake of revisiting a box in which a token had previously been found. The sample’s median percentile was 15.5 (range 3-99), with only 23% (5/22) of the sample in percentile 42 or higher. In total, 36% (8/22) of the participants were in the lower range (percentile 10).

In the RVP task, both the *z* scores of how good the participants were at detecting target sequences (RVP A’, prime; RVP prime, the accuracy to detect correctly the targets [RVPA}) and the probability of false alarm (RVP probability of false alarm; RVPPFA) were relatively low (RVPA: mean –0.5, SD 0.6 and median –0.5, range –1.4 to 1.2; RVPPFA: mean 0.3, SD 0.9 and median 0.1, range –1.5 to 2.3). While the frequency of false alarms was similar to that of the norm (Cohen *d*=0.30; t_21_=1.41; *P*=.17), our participants were slightly worse at detecting the target sequences (Cohen *d*=–0.81; t_21_=–3.80; *P*=.001). Another 14% (3/22) of the participants had a probability of false alarm of 0 (percentile 99). In both measures, only 5% (1/22) of the participants were in a percentile of <10 (8 for the RVPA and 7 for the RVPPFA) who were also in percentile 5 in the SWM task. The sample’s median percentile for the RVPA measure was 29.5 (range 8-89), indicating that half (11/22, 50%) of the sample had equal or worse capability to detect target sequences than the bottom 29.5% in the CANTAB normative data.

Finally, the sample’s mean efficiency in performing the IED task (more detailed information about the outcome measures used in this study can be found in Multimedia Appendix 1) was not different from the norm mean *z* scores (mean 0.0, SD 0.7; median 0.1, range –1.3 to 0.8; Cohen *d*=–0.06; t_21_=–0.28; *P*=.78). However, the participants’ performance was heterogeneous, with a median percentile of 49.6 (IQR 28.8-68.5). In total, 9% (2/22) of the participants were in percentile 9, which indicates that only 9% in the normative data made more errors in this task than these participants.

No correlation was found between the CANTAB *z* scores and any relevant sociodemographic data (ie, sex, age, number of children, failing in school, alcohol and caffeine consumption, years in service, and hours worked per week as a firefighter; sex: CANTAB_SWM: *P*=.81; CANTAB_RVPA: *P*=.499; CANTAB_RVPPFA: *P*=.31; CANTAB_IED: *P*=.50; age: CANTAB_SWM: *P*=.46; CANTAB_RVPA: *P*=.29; CANTAB_RVPPFA: *P*=.49; CANTAB_IED: *P*=.75; number of children: CANTAB_SWM: *P*=.57; CANTAB_RVPA: *P*=.55; CANTAB_RVPPFA: *P*=.73; CANTAB_IED: *P*=.60; failing in school: CANTAB_SWM: *P*=.34; CANTAB_RVPA: *P*=.19; CANTAB_RVPPFA: *P*=.84; CANTAB_IED: *P*=.12; alcohol: CANTAB_SWM: *P*=.37; CANTAB_RVPA: *P*=.61; CANTAB_RVPPFA: *P*=.37; CANTAB_IED: *P*=.17; caffeine consumption: CANTAB_SWM: *P*=.31; CANTAB_RVPA: *P*=.44; CANTAB_RVPPFA: *P*=.66; CANTAB_IED: *P*=.43; years in service: CANTAB_SWM: *P*=.496; CANTAB_RVPA: *P*=.63; CANTAB_RVPPFA: *P*=.84; CANTAB_IED: *P*=.998; hours per week: CANTAB_SWM: *P*=.42; CANTAB_RVPA: *P*=.82; CANTAB_RVPPFA: *P*=.49; CANTAB_IED: *P*=.20).

#### ITC-SOPI Results

The results for the ITC-SOPI scales were as expected. The highest mean was obtained for the Sense of Physical Space scale (mean 3.9, SD 0.8; median 3.8, range 2.9-6.4) followed by the Ecological Validity scale (mean 3.8, SD 0.6; median 3.8, range 2.8-4.8) and the Engagement scale (mean 3.8, SD 0.6; median 3.8, range 2.2-4.7). These scores were higher than the middle point of the scale (3) for all subscales (*P*<.001 for all comparisons).

Finally, the Negative Effects scale had the lowest scores, with a mean of 1.7 (SD 0.6; median 1.7, range 1.0-3.0). This mean, although small, was higher than the lowest possible score of 1 on this scale (Cohen *d*=1.25; t_20_=5.71; *P*<.001). The most frequently mentioned negative effects were “feeling tired” (mean 2.1, SD 1.1; median 2.0, range 1-5) and “feeling disoriented” (mean 2.1, SD 1.0; median 2.0, range 1-4), followed by “ocular fatigue” (mean 2.0, SD 1.0; median 2.0, range 1-4).

#### Correlations Between Measures

At baseline (T0), stress as measured using the DASS-21 correlated positively with the NACM (*r*=0.44; *P*=.04) and AAR (*r*=0.43; *P=*.04) subscales of the PCL-5 but not with the full-scale scores (*r*=0.21; *P*=.34). In addition, the stress scale correlated positively with the negative affect scale of the PANAS at baseline (T0; *r*=0.42; *P*=.049) and after the second control condition (*r*=0.46; *P*=.05). On the other hand, the depression scale of the DASS-21 correlated negatively with the positive affect scale of the PANAS at baseline (T0; *r*=–0.51; *P*=.01) and after each condition (control condition 1: *r*=–0.63 and *P*=.002; experimental condition: *r*=–0.54 and *P*=.009; control condition 2: *r*=–0.70 and *P*<.001). The anxiety dimension of the DASS-21 did not show any correlation with the PANAS for any of the moments measured.

Posttraumatic stress symptoms, as measured using the PCL-5, did not correlate with affect measures (PANAS) except after the arousing experimental condition. Participants who held higher full-scale (*r*=0.43; *P*=.047), intrusion symptom (*r*=0.53; *P*=.01), and avoidance (*r*=0.71; *P*<.001) scores (measured using the PCL-5 at baseline [T0]) had increased negative affect scores measured using the PANAS after the experimental condition. A partial correlation controlling for anxiety, depression, and stress scores (DASS-21) confirmed this result (*r*=0.49 and *P*=.04, *r*=0.51 and *P=*.03, and *r*=0.75 and *P*<.001, respectively). Conversely, even though it appears that there is a negative relation between NACM (as measured using the PCL-5) and positive affect (as measured using the PANAS) after the experimental condition (*r*=–0.48; *P*=.02), this relationship dwindles after controlling for the 3 DASS-21 scales (*r*=–0.45; *P*=.06). A similar result is found when correlating the Avoidance subscale (PCL-5) and the number of adverse events experienced (as measured using the QEPAT). While there appears to be a correlation (*r*=0.44; *P*=.047), it becomes nonsignificant after controlling for the DASS-21 scales (*r*=0.43; *P*=.08).

There was no significant correlation between any of the CANTAB and QASA measures.

### Direct Impact of the VR Training Task

#### Impact on Affect

Performing the VR task had a positive impact on positive affect compared to the baseline (partial η^2^=0.52; Greenhouse-Geisser *F*_1.82, 32.78_=19.73; *P*<.001). Baseline (T0) positive affect was lower than that measured after control condition 1 (*P*=.004), the experimental condition (*P*<.001), and control condition 2 (*P*<.001). The negative affect scores remained consistent throughout the experiment, with no differences found between or after the VR tasks (partial η^2^=0.02; Greenhouse-Geisser *F*_2.13, 38.4_=0.39; *P*=.69). The results found for positive and negative affect remained true after controlling for PCL-5 (full-scale) and DASS-21 scale (depression, anxiety, and stress) scores.

#### Impact on Sense of Presence

The mean results on the ITC-SOPI scales were positive, as mentioned previously. These positive results are highlighted when related to the affect felt throughout the VR tasks and after the experiment ended, controlling for positive and negative affect at T0 (baseline).

When considering the positive affect reported after all VR tasks were completed (ie, at the end of the experiment, after the second control condition) and its relationship to the ITC-SOPI scales, a positive correlation emerges solely with the participants’ reported engagement (*r*=0.55; *P*=.02). Contrarily, the negative affect reported correlated positively and significantly with the negative effects of the VR task as measured using the ITC-SOPI, both after the first nonarousing control condition (*r*=0.61; *P*=.01) and at the end of the experiment (ie, after control condition 2; *r*=0.53; *P*=.03). This is in accordance with the correlation found between the negative effect scale of the ITC-SOPI and other scales. Specifically, a positive and moderate correlation was found between the negative effect and avoidance scores (*r*=0.73; *P*<.001) controlling for baseline levels of depression, anxiety, and stress (DASS-21) and for the number of adverse events experienced (measure obtained using the QEPAT questionnaire). No significant correlations were found between any ITC-SOPI scale and the positive or negative affect reported after the arousing experimental condition. We recognize how idiosyncratic differences in baseline affect could bias results, and therefore, all the aforementioned results do represent partial correlations.

Another noteworthy correlation found was between the number of adverse events that the participants reported having experienced (QEPAT) and the engagement subscale (ITC-SOPI; *r*=0.49; *P*=.02). It appears that participants who experienced more adverse events while in active duty also considered the VR setting more engaging.

#### Impact on Situational Awareness

Regarding the QASA tool, the sample had overall positive results, obtaining a mean rate of correct responses (ie, hit and correct rejections) of 68% (SD 14.6%; median 64.3%, range 43%-93%). This mean rate was similar for the hits (mean 68.2%, SD 21.1%; median 71.4%, range 29%-100%) and correct rejections (mean 67.5%, SD 12.6%; median 71.4%, range 43%-86%). In accordance, the mean rate of incorrect responses was relatively low at 32% (SD 14.6%; median 35.7%, range 7%-57%) for the rate of both misses (mean 31.8%, SD 21.1%; median 28.6%, range 0%-71%) and false alarms (mean 32.5%, SD 12.6%; median 28.6%, range 14%-57%).

Comparably, the mean actual situational awareness, as measured using A’ was relatively high (mean 46.4, SD 34.5; median 45.0, range –25.0 to 92.9), with only 5% (1/22) of the participants having an A′ value of <0 (A′=–25). This indicates that the overall sample had a good situational awareness regarding the elements present in the VR task simulation and was able to distinguish true from false information (with the exception of 1/22, 5% of the participants).

These results were in tandem with the perceived confidence of the participants in their answers (perceived situational awareness [PSA]). The sample’s mean PSA was 47.2 (SD 25.0; median 47.6, range 0-100). All but one participant (21/22, 95%) had confidence in their ability to detect the correct response as they obtained positive values, and only 5% (1/22) of the participants reported not being very confident (ie, PSA value of 0).

When comparing the difference between A′ and PSA scores, it is possible to see that, although no difference between the means was found (mean difference=–0.8; Cohen *d*=–0.02; t_21_=–0.08; *P*=.94), there was a great amount of variation between participants (SD 45.0; median –12.6, range –61.9 to 83.3 in this difference between A’ and PSA). A total of 59% (13/22) of the participants perceived their situational awareness as being higher than it was (reflected by a negative difference), whereas 41% (9/22) had higher situational awareness than they themselves perceived having (as reflected by the positive difference).

The sample presented a slight information acceptance bias, with a mean of –13.6 (SD 30.3; median 0.0, range –100.0 to –9.1). In total, 9% (2/22) of the participants had B″ values of –100, indicating a great tendency to accept information as true even if it was false. A total of 36% (8/22) of the participants did not have any bias as they had a B″ value of 0.

Concordantly with the aforementioned results, no correlation was found between the actual situational awareness (A′) and the PSA (*r*=–0.12; *P*=.59). The same result was found when controlling for experience (ie, years as a firefighter; *r*=–0.11; *P*=.63). PSA values did not correlate with the bias scores (B″; *r*=0.19; *P*=.41) either. The only correlation found was between A′ and B″ scores (*r*=–0.51; *P*=.02) even after controlling for years in service (*r*=–0.50; *P*=.02).

A summary of the main findings of this study can be found in Multimedia Appendix 5.

## Discussion

### Principal Findings

This study showed the viability of a VR firefighter training tool as a valid serious game approach to act as an alternative for in-person real-life training, which can endanger a firefighter’s life and is subject to limitations in the training situations available. Furthermore, the VR setup used in this study allows for ecologically valid and immersive scenarios, which is an advantage for realistic training that prepares for real-life situations.

Participants’ level of acceptability is a crucial aspect to consider for VR use. Many reasons can explain the low tendency to integrate VR settings, namely, skepticism, vulnerability, insecurity, lack of ease of interaction with the equipment [71], or absence of realism [25]. Kari and Kosa [72] presented an interesting model joining hedonic and utilitarian or inconvenience measures to explain use and acceptance of VR games supported by the participants’ behavioral intention to use that was influenced by curiosity and enjoyment. Another relevant aspect is that perceived ease of use was positively correlated with perceived usefulness, curiosity, enjoyment, and control and negatively associated with discomfort; in turn, utilitarian health and well-being factors were less significant aspects in terms of use of VR game applications.

In this study, the VR task was well received by the participants, in concordance with a previous study using the same VR apparatus [68]. Positive affect increased compared to the baseline just by performing the VR task. This was independent of the type of scenario. That is, it appears that being immersed in the VR setting itself improves positive affect, whether the scenario was neutral and did not require immediate action (preparing the surroundings of a house for a forest fire) or required urgent action and was arousing (putting out a house fire). This is corroborated by the relationship found between positive affect and engagement, both reported at the end of the overall VR task and both yielding highly positive mean scores. This reflects that engagement with the overall VR task increased as positive affect increased, and vice versa. This is true regardless of idiosyncratic differences in baseline positive and negative affect. For VR training to be successful, firefighters have to be engaged with the task, which could be related to positive rather than negative affect. Indeed, a recent systematic review and meta-analysis demonstrated the positive impact of VR environments on positive and negative affect measured using the PANAS [73].

Another aspect of the success of the VR firefighter training is the positive feedback from participants regarding the simulation itself. At the end of the study, participants reported that the simulations were perceived as real and similar to the real world, which was reflected by the high mean scores regarding the sense of physical space and ecological validity, as well as the reported usefulness for training and learning purposes. This adds to the existing literature that has demonstrated how VR increases the ecological validity of experiments [6] and allows for the transfer of skills from VR to real-world scenarios [7-9].

In this study, the realness (measured using the ecological validity scores) of the VR simulations was independent of both the positive and negative affect felt by the participants, which attests to the validity of the simulation being independent of the participant performing it. Similar results were found with VR applied for higher-education purposes in another study. The study showed that the level of immersion is crucial for students’ sense of presence and positive affect (also measured using the PANAS). Our results contribute to the generally accepted premise that VR has a positive impact on users’ learning and training outcomes [73]. In the same vein, Shafer et al [74] presented a model explaining how enjoyment is generated in VR applications. The authors argued that participants’ perceived interactivity predicts spatial presence and realism in the task, whereas realism independently predicts spatial presence and, in turn, spatial presence predicts enjoyment; cybersickness was included as a covariate influencing enjoyment, and previous experience using VR or similar experiences had a weak impact on cybersickness.

Another point to consider regarding the viability of VR firefighting training is the dampening of negative effects. It could be argued that these might surpass positive effects if the use of VR leads to greater distress or discomfort or puts into question the health of the participants. In this study, we found no validation for this tenet. On the contrary, participants’ negative affect remained low at baseline and throughout the VR task, with no impact of the arousing, urgent house fire scenario. These results (demonstrated by the firefighters’ positive feedback and low negative emotions) are congruent with the principal dimensions highlighted in a very recent study—usability, usefulness, fun, and joy—as critical aspects to the adoption and recommendation of VR experiences [75].

Nevertheless, a positive correlation was found between the participants’ avoidance, a symptom of PTSD, and the negative affect reported after the arousing scenario. Similarly, participants who reported experiencing symptoms of avoidance more frequently were also the ones who indicated having felt more negative effects due to the VR simulation. This is true regardless of the number of adverse events experienced and of depression, anxiety, or stress symptomatology. This finding should be further explored to ensure that even firefighters who demonstrate some PTSD symptomatology can perform the VR task and are not vulnerable. In addition, while one might expect that experiencing a greater number of adverse events would deter firefighters from engaging in a VR-simulated firefighting environment, the opposite was found. The participants who reported feeling greater engagement with the VR tasks were those who had experienced an increased number of adverse events. Some explanations can be posited in this regard. Indeed, having experienced stress levels during firefighting activities and confrontation with adversity could lead to greater motivation to improve this state of affairs and mobilize participants to act and manage situations, suggesting specific coping strategies and resilience mechanisms [75]. This topic gains importance with respect to implications for intervention strategies, namely, the relevance of including approaches to boost mechanisms of emotion regulation in firefighters, for example, stress buffer interventions or improving distress tolerance programs, as proposed by Stanley et al [40], who highlighted the positive role played by distress tolerance in occupation stress and suicide risk among 831 firefighters.

Nevertheless, the participants reported feeling scarce negative effects due to the VR task alone. The most frequently mentioned effect was “feeling tired,” which might be attributed to the complex implementation of this study itself. Due to the plurality of physiological and psychological measures taken throughout the VR task, the participants had more equipment on them than they would in other VR training contexts and expended more time than usual between scenarios to complete questionnaires. Moreover, this negative effect can be reduced by not performing several VR scenarios in a single training session, thus diminishing fatigue. Regarding “ocular fatigue,” the second most mentioned negative effect, it is something that is expected to subside as technology improves. As expected, negative *affect* measured after most of the VR tasks (control conditions) was related to the negative *effects* reported, both being minute.

The positive and negative emotional experience found in this study was congruent with the results found in a sample of trainees using a VR training system for fire extinction [25].

To further explore the reliability of VR training, it is relevant to assess firefighters’ performance in this task. For this purpose, the QASA tool was developed. It has a 2-fold objective: it both allows for the assessment of whether firefighters are aware of and attentive to the elements present in the simulation and validates that the firefighters are really attentive to the VR task as if it were a real-life scenario. Participants were successful in this task as they managed to correctly identify most of the elements that were true (ie, hit) and most of the elements that were false (ie, correct rejections). However, this alone does not allow us to infer their awareness of the simulation situation. To assess their actual situational awareness, the emphasis should be on the A′ measure, which can be interpreted as a measure of “an individual’s knowledge of the situation as compared to the ‘ground truth” [63]. The participants’ situational awareness was high, indicating that the overall sample was attentive to the simulation and could identify which statements addressing the simulation were true and which were false. This supports the fact that the participants were paying attention to the simulation. However, were the participants aware of their own situational awareness? Although the sample’s mean PSA was similar to their actual situational awareness, there appears to be no correlation between the two, suggesting that performance self-appraisal is not accurate in this setting. In fact, approximately half (11/22, 50%) of the participants overestimated how good their awareness was, whereas the other half (11/22 50%) underestimated it. This is especially surprising considering how all but one participant (21/22, 95%) were confident that most of their responses were correct, some even indicating that all their responses were correct. This result, although expected, has major implications as awareness of the situation is a necessity when it comes to firefighting. Not being attentive to the details of a situation can be the difference between life and death in a real-life scenario. Finally, most participants (11/22, 50%) showed almost no bias in information acceptance. That is, they were considerate when responding to the questions and did not simply accept all the information provided as being true or false.

Concerning the firefighters’ emotional and cognitive characterization, a few depressive, stress, and anxiety symptoms showed some correlation with other measures. For instance, stress levels were correlated with some PTSD symptoms. Participants who had higher values of NACM and AAR also had higher stress values, which was to be expected. Indeed, in the systematic review conducted by Jones [36], a plurality of studies found high comorbidities in firefighters.

Furthermore, participants with higher stress scores additionally reported increased feelings of negative affect (when the study started, before the VR task, and after the VR task was finished). While anxiety scores appear to not have any correlation with any of the other measures, the depression level reported had an inverse correlation with positive affect throughout the experiment. That is, the more the participants felt depressed, the less positive their emotional state was. However, this seems to reflect more on the participants’ typical emotional state than on the VR task’s success and impact. Nonetheless, our sample showed few indicators of psychopathology (as measured using the DASS-21 and PCL-5), which is in concordance with results from a sample of 312 Portuguese firefighters who reported low scores of depression, anxiety, and stress using the same measure used in this study and high levels of happiness [76]. The mental health of firefighters is an issue of particular relevance [37], which cannot be neglected and is emphasized by the aforementioned findings.

In this regard, serious games applied to interventions have revealed promising results in clinical settings, namely, by promoting treatment of and recovery from serious mental illness [77]. Regarding trauma, a recent study presented a contribution toward showing increasing users’ awareness of psychological trauma, their sense of security, and promotion of seeking specialized help, using a serious game combined with psychological principles as theorical frameworks [78]. Another study using a computer game showed potential to reduce intrusive memories of a traumatic event [79]. Positive results using a VR simulator were exhibited by 2 truck drivers with PTSD [80]. This literature suggests a high potential for PTSD treatment using the innovative elements of VR.

Regarding the participants’ cognitive performance, none demonstrated having any sensorimotor deficits or lack of comprehension, measured using the MOT as recommended [62]. Regarding their performance on the remaining cognitive tasks, the capability to not be swayed by false alarm sequences in the RVP task (RVPPFA measure) and their ability to shift between the intra- and extradimensions of a stimulus (IED test; ie, the ability to discriminate between visual set formation and maintain or shift attention in accordance with environmental clues) were no different than those of the sample norm. On the other hand, on average, the firefighters performed worse on the SWM task (ie, remembering which boxes were previously searched) and the ability to detect rapidly shown number sequences (RVPA measure) during the RVP task.

These measures were explored as they convey cognitive functions that are at play when putting out fires. SWM, strategic planning capacity via trial and error, attention and rapid visual processing, abstract thinking, and cognitive flexibility, as the ability to shift attention between elements of a stimulus following environmental clues through an organized search, are all competencies needed when firefighting as rapid decision-making and problem-solving are required. Although questions could be raised regarding the ecological validity of transposing this concise cognitive assessment to a real-life situation, it also brings into deliberation the need to assess a firefighter’s strengths and vulnerabilities when appointing them to a position in action. Questions of ecology are highlighted by the fact that no correlation was found between any of the CANTAB and QASA measures.

### Comparisons With Prior Work

According to previous work, VR serious game systems have high potential for training purposes, giving the possibility to customize them directly in accordance with the users’ needs [2]. In this study, 2 realistic scenarios (and widely related to the daily duties of firefighters) were selected to ascertain the impact on induced positive and negative affect, on the one hand through neutral and nonurgent duties (preventive tasks) and, on the other hand, through urgent, stressful, and arousing activities, namely, extinguishing a house fire and rescuing victims.

During these tasks, firefighters had the opportunity to practice and develop their skill set (eg, dynamic thinking, stress management, decision-making under pressure, risk assessment, fire control, hose handling technique, and nozzle control) and learn in and experience a safe immersive environment, which might not be possible to replicate in real life. These are advantages of VR compared to traditional training according to several authors [2,10,11]. Other features of these systems demonstrated during this study were the capacity to maintain motivation and user engagement [13]. Indeed, participants reported adequate levels of physical presence, ecological validity, and engagement at the end of the experience and residual negative effects, in accordance with previous studies with Portuguese firefighters [15,35]. Furthermore, while performing the tasks, several firefighters provided additional feedback, which supported the system’s similarity to real-life work, as recommended in the literature [15,23], and reinforces its acceptability and usefulness, in agreement with other studies [72,75]. In addition, emotions, a crucial dimension to consider in VR experiences as argued by Saghafian et al [25], were monitored at baseline, between scenarios, and immediately after, showing consistent effects. Collectively, these results demonstrate the reliability of the experiment.

Regarding cognitive performance, some studies have shown the negative impact of a shift work regimen on cognitive functions, which is frequent among Portuguese firefighters (19/22, 86% of our sample were shift workers). In general, our sample revealed normative performance across most tests, which is in line with the considerations of another study conducted with firefighters [44]. These authors assessed cognitive functions using 3 different tests (paired-associate learning, reaction time, and spatial span) of the CANTAB software in simulated hot conditions and further noted that some of these tasks were probably quite easy for their healthy sample of wildland firefighters.

Finally, another key aspect in the firefighters’ performance was their actual situational awareness and how good they perceived it to be, that is, their performance self-appraisal (a metacognitive ability). As Edgar et al [63] note, the correlation between the actual situational awareness and PSA is inconsistent in the literature, sometimes being negligible, as in this study. They further highlight several studies that show that performance is moderated by how good the participants believe their situational awareness to be independently of whether their assessment is correct. They hypothesize that the inconsistency between studies might be explained by a lag between the measurement of actual situational awareness and PSA. This explanation does not fit this study as both measures were collected in the same moment. Nevertheless, efforts should be made not only to improve the actual situational awareness of firefighters but also to align their perception with their actual awareness.

### Limitations and Future Work

This experiment was conducted for research purposes. Although precise instructions were provided for participants to act exactly as in real-life firefighting, these findings should be viewed with this caveat. Despite the greater levels of ecology and presence revealed by the experiment, fighting a fire and rescuing victims in simulated situations will never be exactly the same as in a real-life situation. In the same vein, although the cognitive dimensions assessed are valuable skills for performing fire duties, some caution is needed when transferring these outputs to real practice as they were obtained through computerized abstract tasks. In addition, considerations should be taken regarding the experimental setting itself. As previously mentioned, the experiment involved performing a complex set of diverse tasks (neuropsychological session followed by 3 moments of immersive VR scenarios with simultaneous measurement of biosignals) requiring that participants wear nonusual equipment (eg, electrocardiography, electroencephalography cap, and respiration band) during task performance and involving a wide range of resources. These constraints may challenge ecological transposition to real firefighting activity and could cause fatigue among participants. Despite this limitation, these were requirements of the experimental design, and pauses between procedures were implemented. However, to reduce this potential concern, future work should consider reviewing the duration of the experiment, including the duration of the simulation.

The impact of the psychological assessment on the participants’ performance on the VR task should also be considered in future work. Although the protocol was designed to avoid bias due to mood-congruent memory recall [54], completing the PCL-5, DASS-21, and CANTAB before the VR task could induce fatigue and negative associations from past experiences and have impacted the participants’ VR experience. It is recommended that this interplay between the psychological assessment and VR task is further explored in future research.

One other aspect not accounted for is the level of experience the firefighters had with VR specifically and gaming in general. In our case, participants had a moderate level of experience with immersiveness. Previous research has found that the level of experience and age of the participants influences their immersion in the VR setting [81]. Work on Outcome Measure instruments and Statistical analysis are reported at the Multimedia Appendix 1 [82-91]. Even though this was not the focus of this study, it is an aspect that could lead to a heterogeneous experience for individuals of different experience levels and, thus, should be further investigated in future research.

A final aspect that is worth mentioning is that many firefighters have stated that within a firefighter brigade, each member has a specific role assigned inside the team when deployed in field operations has a specific role assigned inside the team when deployed in field operations. In future work, this issue might be mitigated by including multiplayer tasks to achieve the most realistic situation, similar to a prototypical VR system using a multiuser training application among maritime firefighters [19]. Collaboration between members, communication skills, and collaborative work could also be addressed even more as target abilities of being a firefighter. These are skills easily promoted by opting for VR training settings [14] rather than in-person methodologies.

### Conclusions

An innovative multicomponent approach was applied to a sample of Portuguese healthy firefighters considering psychological variables and cognitive indicators including situational awareness ability combined with a haptic realistic VR training system and simultaneously coupled with physiological measures. Considering that firefighters are a population at high risk, negative affective states of depression, anxiety, and stress were considered, as well as the severity of PTSD symptoms and adverse events witnessed as a firefighter. This is relevant because Portugal is a country annually ravaged by wildfires, with uncalculated costs at various levels.

Promising results were found as participants were engaged during all tasks, revealing high levels of motivation and acceptance related to the VR software. The ecological validity of both immersive scenarios was supported (irrespectively of facing neutral or arousing stress-inducing conditions), with a positive impact (positive affect was higher after the experiment) that could buffer the minor negative effects reported when PTSD symptom severity and anxiety, stress, and depression were controlled for. In general, the sample demonstrated adequate cognitive abilities and awareness of significant elements of the situations even though they did not correspond exactly with their perception.

These results attest to VR translatability to the real world as a valid cost-effective alternative to traditional in-person training closer to the specificity of firefighter activity. The use of innovative technologies such as the haptic and immersive VR software used in this study was attested for firefighter training and learning purposes and complemented prior work, combining several methodologies in a multicomponent approach. In addition, serious games and VR immersive tools might be put at the disposal of professional corpora to improve work conditions.

Valid contributions to this field of study emerged. Implications for real-world practice are important in relation to the impact of being a firefighter as well as the consequences of daily duties and identification of variables that have to be considered for improving training programs fit to the demands of firefighting work and the daily challenges faced.

## Appendix

Multimedia Appendix 1 Detailed information about the outcome measures used in this study, the internal consistency of the scales, and detailed information about the statistical analysis performed in this study.

Multimedia Appendix 2 Descriptive statistics of scales, Cronbach α values, and detailed information about the duration of the experiment (conditions and resting intervals).

Multimedia Appendix 3 Participants’ detailed sociodemographic data.

Multimedia Appendix 4 Positive and Negative Affect Schedule scores throughout the study (baseline, control condition 1, experimental condition, and control condition 2).

Multimedia Appendix 5 Summary of the main findings of this study.

## Abbreviations

AAR: Alterations in Arousal and Reactivity
CANTAB: Cambridge Neuropsychological Test Automated Battery
DASS-21: 21-item Depression, Anxiety, and Stress Scales
IED: intra/extradimensional set shift
ITC-SOPI: ITC–Sense of Presence Inventory
MOT: motor screening task
NACM: Negative Alterations in Cognitions and Mood
PANAS: Positive and Negative Affect Schedule
PCL-5: Posttraumatic Stress Disorder Checklist for the Diagnostic and Statistical Manual of Mental Disorders, Fifth Edition
PSA: perceived situational awareness
PTSD: posttraumatic stress disorder
QASA: Quantitative Analysis of Situation Awareness
QEPAT: Questionário de Exposição e Perturbação dos Acontecimentos Traumáticos (in Portuguese, a questionnaire related to the exposure to and disturbance of traumatic events)
RVP: rapid visual information processing
RVPA: rapid visual information processing prime, the accuracy to detect correctly the targets
RVPPFA: rapid visual information processing probability of false alarm
SWM: spatial working memory
VR: virtual reality

## Data Availability

The datasets generated during and analyzed during this study are not publicly available due the use of personal psychological evaluation data but are available from the corresponding author on reasonable request.
