# Supplementary material for: Exploring Immersive Multimodal Virtual Reality Training, Affective States, and Ecological Validity in Healthy Firefighters: Quasi-Experimental Study
Source: JMIR Serious Games. 2024 Oct 24;12:e53683. doi: 10.2196/53683 (PMC11544332; doi:10.2196/53683)
Supplement: Multimedia Appendix 1 [file games_v12i1e53683_app1.docx]

### Multimedia Appendix 1

### Detailed information about the outcome measures used in this study, the internal consistency of the scales, and detailed information about the statistical analysis performed in this study.

### Outcome Measures

*Sociodemographic and self-reported medical data.* A semi-structured interview was conducted to collect sociodemographic data, information regarding firefighter activity, and medical history.

*The Positive and Negative Affect Schedule* (PANAS; [1] [2])

The scale measures 20 emotions, comprising two dimensions - *Positive Affect* (PA) and *Negative Affect* (NA). The firefighters had to classify how they felt *at the present moment*, using a scale between 1 (*very* slightly or not at all) and 5 (*very much*), providing information about the participant’s affective state exactly at the time of reporting (*right now*). This temporal instruction was foreseen by the original authors [1]. According to them, PA contemplates the degree in which a person feels “enthusiastic, active, and alert”. Conversely, NA reflects unpleasant moods as “anger, contempt, disgust, guilt, fear, and nervousness”. The score is calculated separately for the PA and NA scales, ranging between 10 and 50. Higher scores indicate higher levels of positive and negative affect at the moment of responding. The PANAS scale was administered before the VR experience (T0 - baseline) and immediately after performing each scenario, in order to measure the mood induced by the experience. Three participants did not perform the last VR setting (second control condition) and therefore did not complete the last PANAS.

*21-item* *Depression, Anxiety, and Stress Scales-* (DASS-21[3] [4])

DASS-21 is a self-reported measure with 21-item assessing anxiety, depression, and stress during the last week, using a four-point Likert scale (0-*did not apply to me at all* to 3-*applied to me very much or most of the time*). The scores range from 0 to 21**,** with higher scores indicate higher emotional levels [4]. In this study, we followed the cut-off scores for conventional severity labels recommended by the authors [5]. Nevertheless, for more details, the manual should be consulted, to avoid misinterpretation, as recommended by the authors.

This measure was completed before the VR experiment.

*Questionário de Exposição e Perturbação dos Acontecimentos Traumáticos* (QEPAT; a Portuguese questionnaire related to the exposure to and disturbance of traumatic events; [6]):

A self-report questionnaire related to the exposure and disturbance of traumatic events with a list of 42 adverse events (with possibility to add another event, non-specified previously) directed at firefighting work. This is based on Carvalho and Maia [6].

For each event, the firefighter should indicate the level of exposure during firefighting service, using a five-point Likert scale (0-*never* to 4-*frequently*) and the level of subjective disturbance caused by the event, using a similar five-point scale (0- *not at all* to 4-*very much*). In this study, all participants were instructed to classify the event’s impact only for the situations witnessed at least once. Additionally, firefighters were asked to select the most impactful event, indicating additional information about the experience, including the level of perceived trauma.

*PTSD Checklist for the Diagnostic and Statistical Manual of Mental Disorders, Fifth Edition* (PCL-5 [7] [8]):

A self-report 20-item scale to evaluate the severity of PTSD-related symptoms, used here to exclude firefighters with suspicion of PTSD, for screening purposes. This scale also allows the assessment of PTSD clusters, according to the Diagnostic and Statistical Manual of Mental Disorders – Fifth edition (DSM-5) [9]: *Intrusion, Avoidance, Negative Alterations in Cognitions and Mood (NACM*), and *Alterations in Arousal and Reactivity (AAR)*.

Participants completed the PCL-5 immediately after the QEPAT, and rated the frequency of each symptom, during the last month, using a five-point Likert scale (0-*not at all* to 4-*extremely*), regarding the most impactful event experience as a firefighter, selected previous in the QEPAT, in an attempt to define a traumatic event criterion restricted to firefighter duties, given the aim of the study (both scales were completed before the VR experience), and ultimately, analyze the presence or absence of PTSD symptoms and their severity, in order to guarantee the participants’ security and remove them from any potential triggering situations.

The scale ranges from 0 to 80, with higher scores expressing higher symptoms’ severity. In this study, the 31 cut-off was used to evaluate the probable presence of PTSD [10]. According to the authors, the scale also allows to define a provisional diagnosis of PTSD, considering items reported as *moderately* or higher (at least one symptom reported from Intrusion and Avoidance dimensions, and two or more symptoms from cluster NACM and AAR).

*Cambridge Neuropsychological Test Automated Battery (CANTAB)* [11]:

Four tests were selected from this computerized cognitive test battery to assess the cognitive performance on a variety of specific cognitive abilities and obtain outputs of relevant skills to firefighting (ie, discrimination of significant stimuli, visual attention and flexibility, visuospatial memory, sustained attention, and executive functions) in order to characterize this sample of healthy firefighters, and to ascertain cognitive functioning.

Two different metrics were used across the four tests: the *z* scores (with a normative mean of 0) and percentile ranks (considering data collected through a web-based cognitive assessment application from a normative sample, available from CANTAB software, by gender, level of education and date of birth; comparison was made against all three demographics). Various tests from this digital cognitive battery have been widely used in research, for example to study cognitive functions of firefighters under hot conditions [12] or military troops [13], as well as executive functions [14] and sustained attention in the presence of PTSD symptoms [15]

The selected tests were administered in this sequence, before VR tasks:

**Motor screening task (MOT),** comprises a set of stimuli (10 colored crosses) as a screening measure for sensorimotor deficits or comprehension difficulties, in which firefighters had to tap the target on the screen, quickly and accurately during the trials.

**Spatial working memory (SWM) task (extended version),** as a measure of executive functioning, specifically visuospatial working memory, ability to retain and manipulate information previously visualized, which addresses lobe frontal functioning [16]. The tests provide a comprehension over the strategy adopted by the firefighters during the trials, as difficulty (number of squares) increases progressively and the participant must select the boxes to find the *token* inside and then transport them to an empty column. An elimination and memorization strategy are required since the instruction given is to not select the same token twice. In this study, the output measures used for this test (labeled as SWMBE468) was the number of errors provided by revisiting a square where a yellow token has been found in a previous search, across all 4, 6, and 8 trials [11].

**Rapid visual information processing (RVP) task (3-target version)**

This task is a measure of sustained attention and consists of three target sequences, which appear randomized on the screen mixed with digits from 2 to 9. This type of task is based on the principles of Signal Detection Theory and decision-making process [15]. Participants must tap the screen quickly when a sequence appears and not tap with other digits. The level of difficulty progressively increases from one to three sequences consecutively. In this study, two metrics were considered: RVPA, as a measure of accuracy to detect correctly the targets (*the true positives*); and RVPPFA, representing the probability of false alarm, as a measure of quality - a proportion which combines the number of times the participant provided correct responses (ie, not tapping the screen when the target sequence is not shown) and incorrect responses (ie, inappropriately choosing to tap the screen when the target sequence is not shown) [11].

**Intra/extra dimensional set shift (IED) (lines-first repeated version)**

This test is similar to the Wisconsin Card Sorting Test (WCST [17]) and measures executive functioning, and more specifically problem-solving ability, trial-error learning, planning, and cognitive flexibility. The test starts with a correct pattern and the participant has to learn the underlying rule to discover the correct response, using the feedback words (c*orrect* or *incorrect*). The level of difficulty progressively increases, from simpler to more complex stimuli. The measure used in this study was: the Total Errors Adjusted (IEDYERTA) - selecting a stimulus incorrectly, which does not follow the current rule, adjusted for every stage that was not completed. It represents a measure of efficiency in the test [11].

*Quantitative Analysis of Situation Awareness* (QASA; instrument created for this study following the methodology proposed by Edgar et al [18]:

This version of the QASA instrument was developed specifically for this study, as it assesses the participants’ awareness of a given situation. It is especially useful in open-world simulated firefighting training situations, such as the one used in the present study, seeing how it provides a measure of performance of the firefighters’ attention to the VR task, which is a distinguishing aspect of being involved in the arduous firefighters’ duties.

This test is composed of two types of scales. The first consists of statements regarding the simulation (only for the EC condition, the arousing scenario), seven of which are true and the other seven are false. The participants had to indicate if they believed the statement to be true or false. The second type was a confidence scale, on which the participants were required to indicate, on a four-point Likert scale (1 to 4), how confident they were on their previous answers concerning the truthiness of the statements.

Given the exploratory open-world nature of the simulated task, special attention was given during construction of the test to include only statements that addressed elements of the simulation that the participant was sure to see. The final test was constructed according to the following procedure: an item matrix and item pool were created; the items which were clearer in their interpretation were selected; an interview with an experienced firefighter was conducted to ensure the adequacy of the items, with adjustments made accordingly; two pilot healthy firefighters conducted the simulation VR task, allowing for betterment of the items; the resulting final version was the one used in the study.

This instrument evaluates actual situational awareness (measured by A’), bias of information acceptance or rejection (measured by B’’), and perceived situational awareness (PSA), following a signal detection theory approach [19]. It is used here as a measure of actual performance regarding the participants’ situational attention awareness, and their ability to detect and recall which elements were present in the simulation, and which were not (ie, the ability to distinguish signal from noise, measured by A’). The test also measures perceived performance (ie, participants’ level of confidence in the accuracy of the recall, measured by PSA). Furthermore, it is possible to assess the participants’ biases regarding the acceptance or rejection of information (measured by B’’, regardless of it being true (ie, signal) or false (ie, noise)). That is, if the participants have a tendency to accept all information as true, even when it is false, or to reject information as false, even when it is true.

As stated by the original authors [18], A’ and B’’ follow the equations described by Stanislaw and Todorov [19], as seen below in (1) and (2), respectively. A’ ranges from 0 to 1, with 0.5 indicating an inability to distinguish signal (ie, the target, correct responses) from noise (ie, the distractors, wrong responses). Following (2), B’’ ranges from -1 to +1, with negative scores indicating a tendency to accept information as true, even if false (increasing acceptance as values approach -1), and positive scores indicating the opposite, a tendency to reject information as false, even if true (increasing rejection/non-acceptance as values approach 1). A B’’ value of 0 indicates the absence of bias. PSA values are obtained by computing the mean of responses to all confidence items, with higher scores indicating higher confidence in ones’ responses. Following recommendation by the authors [18], all measures were rescaled to a scale ranging from -100 to +100. In the following equations, *H* represents the hit rate (ie, number of *true* statements correctly identified as true, divided by total number of questions) and *F* represents the false alarms (ie, number of *false* statements incorrectly identified as true, divided by the total number of questions).

Equation models to calculate actual situational awareness (A’), and bias of information acceptance or rejection (B’’), following a signal detection theory approach [19]


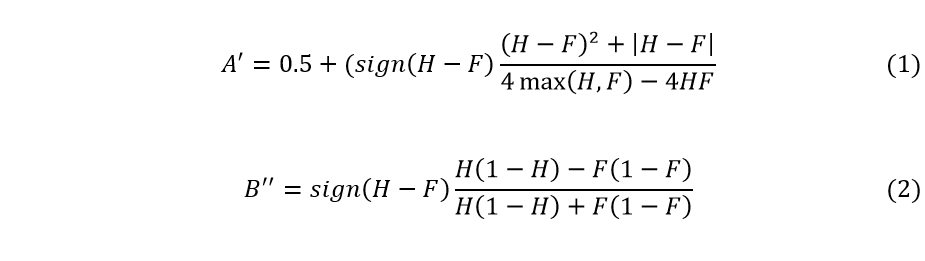


*Note:* A’ – Actual situational awareness, B’’ – Bias of information, H – Hit rate, F – False alarms.

*ITC-Sense of Presence Inventory* (ITC-SOPI [20] [21]):

The instrument includes 35 items to evaluate the participants’ experience after (two questions) and during (33 questions) the VR simulation, in regard to *Sense of Physical Space* (the feeling of being physically present into the simulation and measures the ability to control and manipulate details of the experience), *Engagement* (the level of involvement and interest with the experience), *Ecological Validity* (measures the realism and naturalness of the VR environment), and *Negative Effects* (the subjective reactions caused by the simulation) [21]. Participants responded using a five-point Likert scale, with a range between 1-*strongly disagree* to 5-*strongly agree*), where higher scores represent higher levels of sense of presence during the VR experience.

In this study, the ITC-SOPI was the last filled-in measure immediately after the end of the simulation (CC2 moment), but after the PANAS. For participants’ convenience and comfort, they had already removed all equipment at this time. Each dimension produces a mean score. One participant did not complete the ITC-SOPI, given that they discontinued the experiment before completing the last VR scenario (CC2).

Internal consistency of the scales was generally acceptable, although some obtained low Cronbach alpha values. Alpha was lowest for the *ITC-SOPI Sense of Physical Space scale* (Cronbach α=.32), the *PCL-5 Negative Alterations in Cognitions and Mood subscale* (Cronbach α=.33), and for the *Negative Affect* PANAS, filled out immediately after the first control condition (CC1) (Cronbach α=.49). Alpha values were more heterogeneous in the subscales of PCL-5, ranging between .33 and .94. Detailed alpha values can be found in Table S1 in Multimedia Appendix 2.

### Statistical analysis

Statistical analysis was performed using SPSS (version 28; IBM Corp), and a significance level of 0.05 was established.

Initially, the normality assumption was tested using Kline’s criteria [22]. All measures were within the expected parameters (ie, skewness lower than |3|, kurtosis lower than |10|) and thus parametric analysis was performed. Data on the psychological measures’ skewness and kurtosis are presented in Table S1 in Multimedia Appendix 2. All P values presented are two-tailed.

Statistical analysis was divided into two sections: (1) description of the sample’s values across measures, throughout the experiment; and (2) analysis of the impact the tasks had on the subjects’ psychological landscape.

In the first section, frequency (ie, absolute frequencies, percentages), central tendency (ie, mean, median), and dispersion (ie, standard deviation, range) measures are reported, as well as Cronbach alpha (discussed above). When relevant, means are compared with the minimum, maximum, and intermediate possible values of each scale, using a one-sample t test. This section concludes with the analysis of correlations between measures, using Pearson bivariate and partial correlations.

In the second section, we analyzed the direct impact the VR task had on affect, sense of presence, and the participants’ performance regarding their situational awareness. To test for changes in affect, a Repeated Measures ANOVA was performed, complemented with Bonferroni correction for multiple comparisons. Sphericity assumption was assessed by the Mauchly’s test, and as it was violated, the Greenhouse-Geisser F statistic was reported. To test for the impact of the VR task on the sense of presence, Pearson and partial correlations were conducted. Lastly, participants’ situational awareness was analyzed using the QASA methodology.

All scales' detailed descriptive statistics and Cronbach alphas values can be found in Table S1 in Multimedia Appendix 2. The PANAS and ITC-SOPI presented after the CC2 have an *n* of 19 and 21 respectively, for the reasons previously mentioned.

## References

1. Watson D, Clark LA, Tellegen A. Development and validation of brief measures of positive and negative affect: the PANAS scales. J Pers Soc Psychol. 1988;54(6):1063–1070. doi: 10.1037/0022-3514.54.6.1063

2. Galinha IC, Pais-Ribeiro JL. Contribuição para o estudo da versão portuguesa da positive and negative affect schedule (PANAS): II – Estudo psicométrico. Análise Psicológica 2005;2(XXIII):219–227.

3. Lovibond PF, Lovibond SH. The structure of negative emotional states: comparison of the Depression Anxiety Stress Scales (DASS) with the Beck Depression and Anxiety Inventories. Behaviour Research and Therapy. 1995 Mar;33(3):335–343. doi: 10.1016/0005-7967(94)00075-U

4. Pais-Ribeiro JL, Honrado A, Leal I. Contribuição para o estudo da adaptação portuguesa das Escalas de Ansiedade, Depressão e Stress (EADS) de 21 Itens de Lovibond e Lovibond. Psicologia, Saúde & Doenças. 2004;5(1):229–239.

5. Lovibond SH, Lovibond PF. Manual for the depression anxiety stress scales. 2nd ed. Psychology Foundation of Australia; 1995. ISBN:733414230

6. Carvalho C, Maia Â. Perturbação pós-stress traumático e indicadores de (in)adaptação em bombeiros portugueses. 1.º Congresso de Saúde e Comportamento dos Países de Língua Portuguesa. 2009; Braga, Portugal. CIPSI edições; 2009: p. 277–290. https://hdl.handle.net/1822/11328

7. Weathers FW, Litz BT, Keane TM, Palmieri PA, Marx. B. P., Schnurr PP. The PTSD Checklist for DSM-5 (PCL-5). National Center for PTSD Scale available from the National Center for PTSD. 2013. Available from: https://www.ptsd.va.gov/ [accessed Jul 4, 2023]

8. Silva JCD da. Posttraumatic Stress Disorder Checklist for DSM-5 (PCL-5): validação e invariância da medida numa amostra de bombeiros voluntários portugueses. Dissertation. Universidade do Minho; 2018. http://repositorium.sdum.uminho.pt/bitstream/1822/55699/1/dissertação_final.pdf [accessed Jul 4, 2023]

9. American Psychiatric Association, DSM-5 Task Force. Diagnostic and statistical manual of mental disorders: DSM-5^TM^. 5th ed. American Psychiatric Association; 2013. doi: 10.1176/appi.books.9780890425596ISBN:0-89042-555-8 [accessed Jul 4, 2023]

10. National Center for PTSD. Using the PTSD Checklist for DSM-5 (PCL-5). 2018. Available from: www.ptsd.va.gov [accessed Jul 4, 2023]

11. CANTAB® [Cognitive assessment software]. Cambridge Cognition. All rights reserved. www.cantab.com. 2023 [accessed Jul 4, 2023]

12. Williams-Bell FM, Aisbett B, Murphy BA, Larsen B. The effects of simulated wildland firefighting tasks on core temperature and cognitive function under very hot conditions. Front Physiol. 2017 Oct 24;8(815):1–10. doi: 10.3389/fphys.2017.00815

13. Makhani A, Akbaryan F, Cernak I. Cognitive performance improvement in canadian Armed Forces personnel during deployment. J Mil Veteran Fam Health. 2015 Feb;1(1):59–67. doi: 10.3138/jmvfh.2014-04

14. Olff M, Polak AR, Witteveen AB, Denys D. Executive function in posttraumatic stress disorder (PTSD) and the influence of comorbid depression. Neurobiol Learn Mem.2014 Jul;112:114–121. doi: 10.1016/j.nlm.2014.01.003

15. Zhu H, Li Y, Yuan M, Ren Z, Yuan C, Meng Y, Wang J, Deng W, Qiu C, Huang X, Gong Q, Lui S, Zhang W. Increased functional segregation of brain network associated with symptomatology and sustained attention in chronic post-traumatic stress disorder. J Affect Disord. 2019 Mar;247:183–191. doi: 10.1016/j.jad.2019.01.012

16. Robbins TW, James M, Owen AM, Sahakian BJ, Lawrence AD, McInnes L, Rabbit PMA. A study of performance on tests from the CANTAB battery sensitive to frontal lobe dysfunction in a large sample of normal volunteers: Implications for theories of executive functioning and cognitive aging. Journal of the International Neuropsychological Society. 1998 Sep 1;4(5):474–490. doi: 10.1017/S1355617798455073

17. Heaton RK. A manual for the Wisconsin Card Sorting Test. Odessa, FL: Psychological Assessment Resources; 1981.

18. Edgar GK, Catherwood D, Baker S, Sallis G, Bertels M, Edgar HE, Nikolla D, Buckle S, Goodwin C, Whelan A. Quantitative Analysis of Situation Awareness (QASA): modelling and measuring situation awareness using signal detection theory. Ergonomics. 2018 Jun 3;61(6):762–777. doi: 10.1080/00140139.2017.1420238

19. Stanislaw H, Todorov N. Calculation of signal detection theory measures. Behavior Research Methods, Instruments, & Computers. 1999 Mar;31(1):137–149. doi: 10.3758/BF03207704

20. Lessiter J, Freeman J, Keogh E, Davidoff J. A cross-media presence questionnaire: the ITC-Sense of Presence Inventory. Presence: Teleoperators and Virtual Environments. 2001 Jun;10(3):282–297. doi: 10.1162/105474601300343612

21. Vasconcelos-Raposo J, Melo M, Teixeira C, Cabral L, Bessa M. Adaptation and validation of the ITC - Sense of Presence Inventory for the Portuguese language. Int J Hum Comput Stud. 2019 May 1;125:1–6. doi: 10.1016/j.ijhcs.2018.12.005

22. Kline RB. Principles and practice of structural equation modeling. 4th ed. New York: Guilford Publications; 2016. ISBN: 9781462523344
