# Supplementary material for: Exploring Immersive Multimodal Virtual Reality Training, Affective States, and Ecological Validity in Healthy Firefighters: Quasi-Experimental Study
Source: JMIR Serious Games. 2024 Oct 24;12:e53683. doi: 10.2196/53683 (PMC11544332; doi:10.2196/53683)
Supplement: Multimedia Appendix 2 [file games_v12i1e53683_app2.docx]

**Multimedia Appendix 2**

**Table S1**

*Characterisation of psychological measures’ values*

| **Measures** | | **Mean (SD)** | **Median (range)** | **α** | **Skewness** | **Kurtosis** |
| --- | --- | --- | --- | --- | --- | --- |
| DASS-21^a^ Depression | | 1.1 (1.3) | 1.0 (0-4) | .50 | 1.35 | 1.32 |
| DASS-21^a^ Anxiety | | 1.2 (1.5) | 0.5 (0-5) | .57 | 0.99 | -0.01 |
| DASS-21^a^ Stress | | 3.7 (2.6) | 3.5 (0-8) | .68 | -0.02 | -1.10 |
| PCL-5^b^ Full Scale | | 3.9 (4.5) | 2.0 (0-12) | .75 | 0.98 | -0.20 |
|  | PCL-5^b^ Intrusion Symptoms | 1.0 (1.5) | 0.0 (0-5) | .59 | 1.86 | 3.43 |
|  | PCL-5^b^ Avoidance | 1.0 (1.9) | 0.0 (0-8) | .94 | 1.58 | 1.50 |
|  | PCL-5^b^ NACM^c^ | 1.1 (1.7) | 0.0 (0-6) | .33 | 1.17 | 0.02 |
|  | PCL-5^b^ AAR^d^ | 0.9 (1.3) | 0.0 (0-4) | .56 | 1.51 | 1.14 |
| QEPAT^e^ Number of Events | | 31.9 (4.5) | 33.0 (23-40) | - | -0.28 | -0.52 |
| PANAS^f^ T0^g^ | |  |  |  |  |  |
|  | Positive Affect | 32.4 (7.2) | 33.5 (19-44) | .86 | -0.33 | -1.08 |
|  | Negative Affect | 11.2 (1.6) | 11.0 (10-17) | .61 | 2.57 | 8.17 |
| PANAS^f^ Control Scenario 1 | |  |  |  |  |  |
|  | Positive Affect | 38.0 (7.0) | 39.0 (19-50) | .86 | -0.25 | -0.10 |
|  | Negative Affect | 11.0 (1.3) | 10.0 (10-14) | .49 | 1.01 | -0.49 |
| PANAS^f^ Experimental Scenario | |  |  |  |  |  |
|  | Positive Affect | 40.0 (6.8) | 41.0 (17-49) | .85 | -0.13 | 0.02 |
|  | Negative Affect | 11.9 (3.2) | 10.0 (10-24) | .81 | 1.21 | 0.36 |
| PANAS^f^ Control Scenario 2 | |  |  |  |  |  |
|  | Positive Affect | 40.7 (6.6) | 43.0 (22-50) | .86 | -1.37 | 2.45 |
|  | Negative Affect | 11.0 (1.8) | 10.0 (10-16) | .74 | 1.81 | 2.53 |
| ICT-SOPI^h^ | |  |  |  |  |  |
|  | Sense of Physical Space | 3.9 (0.8) | 3.8 (2.9-6.4) | .32 | 1.74 | 4.45 |
|  | Engagement | 3.8 (0.6) | 3.8 (2.2-4.7) | .68 | -1.20 | 1.78 |
|  | Ecological Validity | 3.8 (0.6) | 3.8 (2.8-4.8) | .75 | -0.10 | -0.52 |
|  | Negative Effects | 1.7 (0.6) | 1.7 (1.0-3.0) | .76 | 0.49 | -0.50 |
| CANTAB^i^ | |  |  |  |  |  |
|  | SWMBE468^j^ | -0.6 (1.3) | -1.0 ((-1.9)-2.3) | - | 1.40 | 0.71 |
|  | RVPA^k^ | -0.5 (0.6) | -0.5 ((-1.4)-1.2) | - | 1.35 | 2.98 |
|  | RVPPFA^l^ | 0.3 (0.9) | 0.1 ((-1.5)-2.3) | - | 1.94 | 3.86 |
|  | IEDYERTA^m^ | 0.0 (0.7) | 0.1 ((-1.3)-0.8) | - | -0.83 | -0.35 |
| QASA^n^ | |  |  |  |  |  |
|  | A’^o^ | 46.4 (34.5) | 45.0 ((-25.0)-92.9) | - | -0.43 | -1.11 |
|  | B’’^p^ | -13.6 (30.3) | 0.0 ((-100.0)-9.1) | - | -2.17 | 4.06 |
|  | PSA^q^ | 47.2 (25.0) | 47.6 (0-100) | - | 0.21 | 0.12 |
| Duration of the experiment^r^ | | 34.1 (8.6) | 33.9 (24.3-47.0) |  |  |  |
|  | Control Condition 1^r^ | 5.7 (0.5) | 5.5 (4.9-7.3) |  |  |  |
|  | Resting interval 1^r^ | 4.3 (3.3) | 3.3 (2.2-17.3) |  |  |  |
|  | Experimental Condition^r^ | 13.8 (3.8) | 13.8 (5.0-20.5) |  |  |  |
|  | Resting interval 2^r^ | 6.5 (1.6) | 5.7 (4.4-8.9) |  |  |  |
|  | Control Condition 2^r^ | 5.4 (0.2) | 5.4 (5.2-5.8) |  |  |  |

^a^ 21-item-Depression Anxiety and Stress Scales; ^b^PTSD Checklist for the DSM-5; ^c^PCL-5 subscale of Negative Alterations in Cognitions and Mood; ^d^PCL-5 subscale of Alterations in Arousal and Reactivity; ^e^Questionnaire related to the exposure and disturbance of traumatic events; ^f^Positive and Negative Affect Schedule; ^g^Baseline measure; ^h^ITC-Sense of Presence Inventory; ^i^Cambridge Neuropsychological Test Automated Battery; ^j^CANTAB Spatial Working Memory measure; ^k^CANTAB Rapid Visual Information Processing, measure of accuracy to detect the targets correctly (*z* scores); ^l^CANTAB Rapid Visual Information Processing, measure of the probability of false alarm as quality of performance (*z* scores); ^m^CANTAB Intra-Extra Dimensional Set Shift measure (*z* scores); ^n^Quantitative Analysis of Situation Awareness; ^o^Actual Situational Awareness; ^p^Bias of Information Acceptance or Rejection; ^q^Perceived Situational Awareness; ^r^Duration is presented in minutes.
