# Supplementary material for: Exploring Immersive Multimodal Virtual Reality Training, Affective States, and Ecological Validity in Healthy Firefighters: Quasi-Experimental Study
Source: JMIR Serious Games. 2024 Oct 24;12:e53683. doi: 10.2196/53683 (PMC11544332; doi:10.2196/53683)
Supplement: Multimedia Appendix 3 [file games_v12i1e53683_app3.docx]

**Multimedia Appendix 3**

**Table S2**

*Participants’ sociodemographic characteristics, consumption habits, Professional status and Firefighting occupation*

| **Characteristics** | | ***f*** | **Valid %** | **N** |
| --- | --- | --- | --- | --- |
| ***Sociodemographic*** | | | |  |
| *Age* (mean 39.1, SD 9.7; median 42.0, range 23-56) | | |  | 22 |
| *Sex* | |  |  | 22 |
|  | Female | 7 | 32 |  |
|  | Male | 15 | 68 |  |
| *Nationality* | |  |  | 22 |
|  | Portuguese | 21 | 95 |  |
|  | Other | 1 | 5 |  |
| *Education* | |  |  | 22 |
|  | Primary (7º, 8º, 9º) | 2 | 9 |  |
|  | Secondary (10º, 11º, 12º) | 17 | 77 |  |
|  | Higher education (university) | 3 | 14 |  |
| *Location of residency* | |  |  | 22 |
|  | Predominantly urban | 11 | 50 |  |
|  | Predominantly rural | 7 | 32 |  |
|  | Mixed urban and rural | 4 | 18 |  |
| *Marital status* | |  |  | 22 |
|  | Single | 9 | 41 |  |
|  | Married | 4 | 18 |  |
|  | Non-marital Union | 5 | 23 |  |
|  | Divorced | 4 | 18 |  |
| *Household size* | |  |  | 22 |
|  | 1 person (self) | 3 | 14 |  |
|  | 2 people | 1 | 5 |  |
|  | 3 people | 8 | 36 |  |
|  | 4 people | 7 | 32 |  |
|  | 5 people | 3 | 14 |  |
| *Number of children* | |  |  | 20 |
|  | 0 | 7 | 35 |  |
|  | 1 | 3 | 15 |  |
|  | 2 | 6 | 30 |  |
|  | 3 | 3 | 15 |  |
|  | 4 | 1 | 5 |  |
|  |  | | |  |
| ***Consumption habits*** | | | |  |
| *Alcohol* | | | | 22 |
|  | Drinks alcohol | 20 | 91 |  |
|  | Does not drink alcohol | 2 | 9 |  |
| *Coffee* | |  |  | 22 |
|  | Drinks coffee | 18 | 82 |  |
|  | Does not drink coffee currently | 2 | 9 |  |
|  | Never drank coffee | 2 | 9 |  |
| *Number of daily coffee cups* (mean 3.9, SD 3.1; median 3.0, range 1-12) | | | | 22 |
| *Smoking* | |  |  | 22 |
|  | Smokes regularly | 8 | 36 |  |
|  | Does not smoke | 9 | 41 |  |
|  | Former smoker | 5 | 23 |  |
|  |  |  |  |  |
| ***Self-reported medical data*** | | | |  |
| *Has required psychological assistance in the past* | |  |  | 22 |
|  | Yes | 13 | 59 |  |
|  | No | 9 | 41 |  |
|  | | | |  |
| ***Firefighting Activity*** | |  |  |  |
| *Work regimen* | |  |  | 22 |
|  | Professional | 12 | 55 |  |
|  | Voluntary | 10 | 45 |  |
| *Years in service* (mean 18.9, SD 10.6; median 18.0, range 4-40) | | | | 22 |
| *Hours worked per week* (mean 36.8, SD 17.5; median 42.0, range 7-60) | | | | 22 |
| *Shift work* | |  |  | 22 |
|  | Does not do shift work | 3 | 14 |  |
|  | Does shift work | 19 | 86 |  |
