# Supplementary material for: Exploring Immersive Multimodal Virtual Reality Training, Affective States, and Ecological Validity in Healthy Firefighters: Quasi-Experimental Study
Source: JMIR Serious Games. 2024 Oct 24;12:e53683. doi: 10.2196/53683 (PMC11544332; doi:10.2196/53683)
Supplement: Multimedia Appendix 4 [file games_v12i1e53683_app4.docx]

**Multimedia Appendix 4**

**Figure S1**


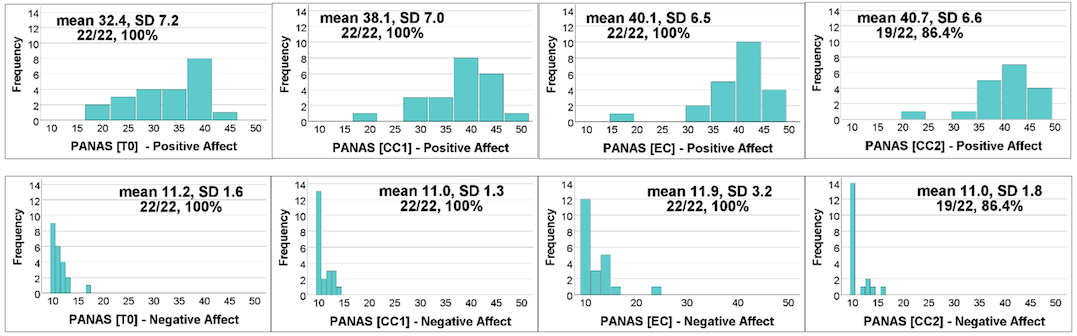


**Figure S1.** Positive and Negative Affect Schedule (PANAS) scores throughout the study (baseline [T0], control condition 1 [CC1], experimental condition [EC], and control condition 2 [CC2]).
