# Supplementary material for: Exploring Immersive Multimodal Virtual Reality Training, Affective States, and Ecological Validity in Healthy Firefighters: Quasi-Experimental Study
Source: JMIR Serious Games. 2024 Oct 24;12:e53683. doi: 10.2196/53683 (PMC11544332; doi:10.2196/53683)
Supplement: Multimedia Appendix 5 [file games_v12i1e53683_app5.docx]

**Multimedia Appendix 5**

**Table S3**

*Summary of Findings*

| **Variables of Interest** | **Main Findings** |
| --- | --- |
| Affective State | Virtual Reality increased positive affect, keeping negative affect low and constant throughout the training. |
| Effects of VR | Engagement with the task was high, with few negative effects. Participants reported the VR setting provided the sense of being in a physical space and had high ecological validity. Engagement was highest for firefighters who had previously experienced more adverse events while in active duty. |
| Situational Awareness | The firefighters had good situational awareness of which elements were present in the VR setting (and which were not), although they had a slight inclination to accept that the elements were present, at face value. Some participants overestimated how good their awareness was, while others underestimated it. |
